# Is the Apple Vision Pro the Ultimate Display? A First Perspective and Survey on Entering the Wonderland of Precision Medicine

**DOI:** 10.2196/52785

**Published:** 2024-09-18

**Authors:** Jan Egger, Christina Gsaxner, Gijs Luijten, Jianxu Chen, Xiaojun Chen, Jiang Bian, Jens Kleesiek, Behrus Puladi

**Affiliations:** 1 Institute for Artificial Intelligence in Medicine Essen University Hospital (AöR) Essen Germany; 2 Center for Virtual and Extended Reality in Medicine (ZvRM) Essen University Hospital (AöR) Essen Germany; 3 Cancer Research Center Cologne Essen (CCCE) University Medicine Essen (AöR) Essen Germany; 4 Department of Oral and Maxillofacial Surgery & Institute of Medical Informatics University Hospital RWTH Aachen Aachen Germany; 5 Institute of Medical Informatics University Hospital RWTH Aachen Aachen Germany; 6 Institute of Computer Graphics and Vision Graz University of Technology Graz Austria; 7 Leibniz-Institut für Analytische Wissenschaften (ISAS) Dortmund Germany; 8 Institute of Biomedical Manufacturing and Life Quality Engineering, State Key Laboratory of Mechanical System and Vibration School of Mechanical Engineering Shanghai Jiao Tong University Shanghai China; 9 Institute of Medical Robotic Shanghai Jiao Tong University Shanghai China; 10 Health Outcomes and Biomedical Informatics College of Medicine University of Florida Gainesville, FL United States; 11 German Cancer Consortium (DKTK) Partner Site Essen Essen Germany; 12 Department of Physics TU Dortmund University Dortmund Germany

**Keywords:** Apple Vision Pro, mixed reality, augmented reality, virtual reality, health care, mobile phone

## Abstract

At the Worldwide Developers Conference in June 2023, Apple introduced the Vision Pro. The Apple Vision Pro (AVP) is a mixed reality headset; more specifically, it is a virtual reality device with an additional video see-through capability. The video see-through capability turns the AVP into an augmented reality (AR) device. The AR feature is enabled by streaming the real world via cameras on the (virtual reality) screens in front of the user’s eyes. This is, of course, not unique and is similar to other devices, such as the Varjo XR-3 (Varjo Technologies Oy). Nevertheless, the AVP has some interesting features, such as an inside-out screen that can show the headset wearer’s eyes to “outsiders,” and a button on the top, called the “digital crown,” that allows a seamless blend of digital content with the user’s physical space by turning it. In addition, it is untethered, except for the cable to the battery, which makes the headset more agile, compared to the Varjo XR-3. This could actually come closer to “The Ultimate Display,” which Ivan Sutherland had already sketched in 1965. After a great response from the media and social networks to the release, we were able to test and review the new AVP ourselves in March 2024. Including an expert survey with 13 of our colleagues after testing the AVP in our institute, this Viewpoint explores whether the AVP can overcome clinical challenges that AR especially still faces in the medical domain; we also go beyond this and discuss whether the AVP could support clinicians in essential tasks to allow them to spend more time with their patients.

## Introduction

In his highly influential paper “The Ultimate Display” [1], Ivan Sutherland, the pioneer of computer graphics and creator of the first augmented reality (AR) head-mounted display (HMD) in the early 1960s, predating the advent of PCs, described aspects that Apple’s Vision Pro aims to implement. The Apple Vision Pro (AVP) can span the wide range of the reality-virtuality continuum, as explained by Milgram et al [2], starting from unaltered *reality*, achieved by streaming the real world without additional information to the user; progressing through *mixed reality* (MR) [3], involving the integration of digital information into the real world (referred to as AR) and vice versa (augmented virtuality); and extending to complete immersion, also known as virtual reality (VR), where the user experiences only computer-generated virtual content.

In the realm of VR, there is currently a broader selection of headsets at various price points, including offerings from Meta, Vive, Varjo, PlayStation, and Google (with the most economical probably being the now-discontinued Google Cardboard or a similar variant from another vendor), compared to AR. AR faces a distinct challenge because of the need to analyze *reality* to augment it at the *right* position with digital content for the user. An exception is AR used for a *pure* simulation, where the AR hologram is indeed shown in the real world but has no meaningful relation to or interactions with real-world objects. An example of such a scenario is a surgical simulation [4]. The AR hologram is shown in front of the user to allow them to inspect and interact with it. Still, the underlying hardware and software need to capture and model the real world to *anchor* the hologram within it so that the hologram does not *drift* away from the user’s view.

In a medical scenario, AR can enable equips a physician with x-ray vision into a patient [5]. This capability is a valuable advancement from the current practice of viewing patient information, such as preoperative imaging, on PC monitors, contributing to the “switching focus problem,” where a physician must divide their attention between the patient and the digital information. Hansen et al [6] outlined that such a division leads to an increased mental workload, disorientation, and deteriorated hand-eye coordination. Instead, AR can display patient information directly in the physician’s view of the patient, additionally offering spatial perception even before any incisions are made. A recent systematic review of the AR headset HoloLens (Microsoft Corp) in medicine [7] showed its broad applications and the massive research efforts during the recent years in all these areas (medicine and health care with applications for patients, physicians or surgeons and students, which should ultimately lead to the development of an intelligent health care metaverse [8,9]). Hence, we highlight the user groups and applications targeted by AR in the next sections and discuss how common challenges in AR can be overcome with the AVP’s capabilities. Furthermore, we share our findings about the new AVP gathered through a structured survey among ourselves and colleagues who had the opportunity to try out the AVP.

## Target Groups

### Physicians and Health Care Professionals

Physicians and health care professionals comprise, by far, the largest target group for AR applications [7]. The adoption and use cases of AR within nursing departments are growing, focusing on wound care, information retrieval, injections, and education [10]. In this context, the accuracy of the visualization is not of utmost importance, but having a wearable “sensing” computer can increase time at the bedside through automatic information retrieval and documentation. However, especially for AR-supported image guidance and navigation, such as in surgery, very high accuracy and reliability are needed [11]. Applications for which submillimeter precision is not necessary are, for example, ablations, ventriculostomies [12-16], and certain orthopedic interventions [17]. Here, the HoloLens is, with its inside-out tracking, already a promising tool, but for applications that need, for example, submillimeter precision, it cannot be used reliably yet. An example is the deep brain stimulation procedure used for treating essential tremors and Parkinson disease, where millimeter-to-submillimeter accuracy in deep brain stimulation targeting (an electrode placement inside the brain) can be important [18]. Another study exploring the clinical accuracy of the HoloLens for neuronavigation concluded that it is currently not within clinically acceptable levels [19]. The same holds true for some application scenarios in orthopedic surgery [20], such as screw placement, where there is still room for improvement [21]. This improvement can be achieved through external tracking hardware [22,23], but it may come at the cost of mobility and increased expenses. The AVP is promising due to its superior sensing technology, as discussed in the Accuracy and Reliability subsection. More importantly, the powerful Apple M1 and R1 chipsets can handle the sensor data appropriately to exploit them completely. This technology comes at a high price, but the often-criticized end-user price of US $3499 (without tax) for the AVP does not seem to be an issue for health professionals who want to use it in health care. The price is similar to that of the HoloLens and much cheaper than (a fraction of) those of existing and clinically used conventional medical navigation systems, for example, those from Brainlab (Stefan Vilsmeier) or Medtronic (Earl Bakken and Palmer Hermundslie). In this context, it is important to mention that the use of AR HMDs with direct patient reference, outside of research in the context of feasibility studies, must obtain appropriate regulatory approval, and the AT HMDs must be certified and classified as medical devices. The Magic Leap 2 (Rony Abovitz), for example, received certification for use in an operating room last year (2023). The requirements to be met are still up to debate, for example, when it comes to active or passive cooling. In contrast to the HoloLens 2, both AVP and Magic Leap have active cooling, potentially compromising the sterile airflow. Furthermore, sterilization is a problem because Apple recommends not using isopropyl alcohol or other solvents for cleaning. This should not be an absolute impediment, given the approval of the Magic Leap 2 for use in the operating theater and the use of regular glasses in the operating theater. We do, however, think that the time is coming when clinicians, technicians, and manufacturers should come together to meet binding guidelines.

Another challenge for clinical adoption is the integration of the device within the digital infrastructure of the hospital, for example, to retrieve data from and transfer data to the electronic health records. Moreover, and especially in a medical context, the display may also require an image focus at surgical table distance [24]. In combination with the outward sensors, this would be ideal for medical scenarios.

### Students

Students are the second most common intended target group [7] for AR applications, for example, in educational training, such as the HoloPointer, a virtual AR pointer for laparoscopic surgery training [25]. Another example is the use of AR to teach medical students catheter placement [26] and the use of AR for training on central venous access [27]. Notably, the aforementioned partially outward-oriented sensors, unlike those in other headsets, allow for the accurate tracking of all “normal” (hand) movements for interaction, regardless of (head) position. This is also valuable for health care professionals when practicing or working at the patient’s bedside. When combined with speech commands and eye-controlled interactions, physicians can expectantly implement the AVP easily and in a sterile manner.

However, we found that the effects of HoloLens-based learning compared to those of conventional learning, for example, using cadavers or other computerized methods, seem to be rather small [7]. A reason for this is that the learning tools usually used are relatively simple, conventional 3D models, and more innovative visualizations, including interactive, dynamic content, which cannot be easily delivered through regular computerized methods, have not been explored in depth yet [7]. Here, we believe that the AVP can raise the bar because of its high-resolution visualization (consisting of 2 micro-OLED displays with 23 megapixels) that uses eye-tracking to make the headset also usable as a desktop screen with its fine textual details. In addition, the 3D user interface, using precise and intuitive finger tracking in combination with eye interactions, can be a game changer. Despite these advances, it remains to be seen whether an AR headset has a major advantage over a conventional screen. The cost of the AVP is also a concern, especially for students with a limited financial budget, but the “pro” might indicate that a “regular” version will also be released. For medical simulation training, however, an examination of costs [28] suggests that using the AVP is a financially viable option. The AVP is, however, highly personalized to the individual user to optimize the experience and accuracy. Luckily for the student group, the AVP can be shared, and the interpupillary distance setting (IDP) is adaptable, compromising the experience minimally, but sharing greatly reduces cost for simulation purposes.

### Patients

Patients are currently the least frequented target group for extended reality (XR) devices [7]. An example is the use of MemHolo, which provides MR experiences for patients with Alzheimer disease [29]. Other examples in this area include the use of the HoloLens as an assistance and monitoring tool for medication adherence [30] and the use of a HoloLens-based system for functional mobility assessment [31]. We expect the advanced eye-tracking to enhance the user experience because visual cues will not merely exist within the user’s field of view but can also be precisely situated in the user’s focal point. However, many interesting assistance and monitoring applications are limited by the restricted possible use time of untethered AR headsets, primarily due to battery life, and this problem seems to exist in the AVP, which has a similar battery life to that of the HoloLens, approximately 2 hours. The only consolation is the “external” battery pack of the AVP, which can be swapped. Finally, the Apple M2 chip and the new R1 chip of the AVP could potentially boost the relatively small number of applications that have been explored so far, for example, by increasing the frames per second, minimizing display latency, and thus reducing motion sickness, especially useful for older patients, who are more susceptible to MR sickness than younger users [32,33].

### Differences Between Target Groups

An example of different use cases per user group is shown in Textbox 1. The main target group is health care professionals, which can be divided into several subgroups, such as surgeons, physicians, and nurses. Unlike the other groups, price is not a major issue for this group, but the use cases are different from those of the other groups. For nurses, data retrieval, documentation, and guidance without the need for high accuracy are the estimated use cases, freeing up time at the bedside. The AVP’s high data transfer rate and processing power are more important. For a surgeon, however, accuracy is critical for surgical navigation [34]. For the general practitioner, seamless integration and unparalleled view during transport may be the greatest values of the AVP. For other groups, price is an important consideration, with the caveat that it could reduce overall costs for medical simulation training. The average patient is not expected to be able to afford this device on their own. For now, it could be used for therapies in the clinic, where it can be shared among several patients. However, this makes cleaning the device more important than if there is a single user. We expect to see silicon-replaceable parts for the device, which would ultimately benefit all groups. The AVP has similar challenges to other headsets in terms of battery life and motion sickness, which affects all groups, especially nurses during long, hectic shifts with movement and sometimes no breaks. The integration of an external battery makes battery life easy to address in future hardware updates. Regarding motion sickness, we expect Apple to offer a lower resolution for a higher refresh rate in combination with its foveated rendering to combat this. In summary, the biggest differences between target groups are in the need for high accuracy and the willingness to accept the high price.

**Possible health care scenarios with the Apple Vision Pro.**

**Health care professionals**

- Patient record retrieval
- Automated documentation
- Guidance in medical procedures and equipment
- Surgical preoperative planning
- Surgical navigation
- Peerless public medical record viewer

**Students**

- Complex anatomy education
- Complex pathophysiology education
- Training simulators
- Automated skill evaluation

**Patients**

- Patient treatment preparation
- Patient education
- (Automated) diagnostics
- Rehabilitation
- Improved patient well-being

## Challenges

Despite significant research efforts in medical AR, clinical translation remains limited so far [35]. Several technical challenges require ongoing multidisciplinary efforts among software developers, hardware manufacturers, regulatory entities, and clinical personnel to create safe, effective, and seamlessly integrated AR solutions in health care. Some of these challenges could be addressed by the AVP’s hardware and user interface.

### Accuracy and Reliability

The most significant challenge in medical AR systems is their accuracy and reliability in registration and calibration, which are critical for aligning virtual objects with real-world elements. Failures in tracking or registration components can misguide physicians, potentially leading to serious medical accidents. Although recent developments in tracking technology are encouraging [36,37], clinical settings pose additional challenges with limited space, unconventional lighting, and dynamic environments. A solution is to combine an AR device with external tracking systems to increase precision [22]. On the downside, such combinations require complicated setups and the calibration of bulky (external) devices, diminishing the simplicity and lightweight nature of purely head-worn systems. The latest sensor technology, combined with state-of-the-art tracking and registration algorithms, could eliminate the need for external systems to meet clinical requirements. We expect the AVP to move the needle in terms of accuracy because of its strong inside-out tracking through 12 built-in cameras and light detection and ranging sensing method, which is the key aspect for increasing the accuracy of AR. We expect that enhanced eye-tracking capabilities and the incorporation of multiple outward-facing sensors will substantially enhance the reliability and stability of user interactions. The increase in both the quantity and range of sensors, coupled with a more potent chipset, is bound to increase spatial resolution and visualization accuracy significantly in comparison to untethered alternatives. Future research should focus on exploiting this improved hardware setup.

### Usability and Human Factors

In complex clinical environments, a seamless integration of AR systems into existing clinical processes is crucial. Existing AR solutions often add undue complexity and disrupt established workflows, requiring intricate hardware, many manual steps, and elevated technical knowledge and understanding. Health care professionals, who possess varying degrees of technical background, or patients, who represent diverse demographics, cannot be reasonably expected to meet these demands. Hence, AR systems need to be intuitive and user-friendly. Apple products are known for their user-centric design and seamless integration of hardware and software. We expect that the AVP’s user experience, in terms of interface, interaction, and feedback, will be more intuitive for a diverse audience than existing HMDs and VR glasses.

As a demonstration of this ease of use, the AVP connects automatically to nearby Apple MacBooks, commonly used by physicians, to display the screen and access files in real time without any effort. An obvious use case is secure, peerless access to patient files while using public transportation. This is valuable for working with all medical records. Apple products work seamlessly with each other, but in a clinical setting, not all products may come from Apple or its partners. A combined challenge and focus should be to enable cross-manufacturer integration.

In addition, as mentioned earlier, more natural and user-guided interactions are possible with the outward and eye-tracking sensors. This will allow developers to create more intuitive applications. The improved hardware should not be underestimated. For example, Gsaxner et al [38] developed stand-alone marker-less image registration, which has potential in many medical applications, but the setup of an additional laptop is cumbersome and another potential risk for data breach. With the AVP, this type of setup is most likely not needed.

### Visualization and Perception

Current AR devices face limitations in terms of view, resolution, and brightness, impacting visual fidelity [39]. In addition to these hardware constraints, challenges include depth perception and occlusion between virtual and real content [40]. Current optical see-through (OST) displays, such as that of the popular HoloLens, struggle to address these issues adequately, as they cannot modify the reality presented. By contrast, the AVP’s video see-through (VST) system provides significantly more possibilities for seamlessly and adaptively integrating virtual elements with the real environment. Visual accuracy is difficult to achieve and user dependent, but eye-tracking and automatic eye calibration, combined with the high-resolution displays inside the headset, make us believe that the AVP could set new standards in terms of the quality and perception of visualization.

In summary, hardware limitations are the main bottleneck in developing AR applications suitable for the clinical translation of AR that cannot be addressed by software developers. Another bottleneck is the regulation and list of requirements for 3D smart glasses in surgery. Combining hardware technology and software from different companies, especially for data transfer, is the final major issue, however, but it is not limited to 3D smart glasses and has been a persistent obstacle in health care [41].

We believe that the AVP can provide significant advancements as the new state-of-the-art device, with much-needed improvements in precision, reliability, usability, workflow, and perception. The combination of AR and VR in an XR device has the potential to transform health care by aiding in diagnostics, improving surgical procedures, facilitating remote patient care, and enhancing medical training. Many other novel applications may emerge because of the advancements in AVP hardware and software platform. Returning to our main question, the AVP has the potential to enhance medical education and therapy, medical record evaluation, data retrieval, and documentation automation while improving patient outcomes and freeing up clinicians to spend more time at the patient’s bedside. Of course, this will require a collaborative effort among engineers, researchers, and clinicians to develop or enhance these applications and to prove cost-effectiveness to ensure clinical adoption.

## The New AVP

### Highlighted Features

Apple announced some interesting (and still unique) features of the AVP. In the upcoming sections, we introduce and discuss these features in the context of medical MR with a focus on AR.

#### Digital Crown

The digital crown is a turning button that allows the user to blend digital content easily and seamlessly with the physical environment surrounding them. The button is named the same as that in the Apple Watch, but it serves a different function in the AVP by providing an easy option to quickly *escape* VR without the need to remove the headset. Depending on the capabilities and accessibility of developers to exploit the digital crown, it could also enable diminished reality (DR) [3,42]. This feature would allow users, such as physicians, students, and patients, to remove distracting real-world objects or environments, thereby reducing visual overstimulation. Notably, DR may even extend to diminishing or removing the headsets of other users. To elaborate, the AVP can create realistic avatars of its users. When using the FaceTime application, the movement and direction of the eyes, tongue, and facial muscles are correctly tracked and visualized for other users of the AVP inside the same FaceTime application. This could be used to give the wearer of the headset the ability to perform DR, that is, to diminish the headset and in paint the face when communicating with other AVP users in the real world but via the pass-through mode. The idea of DR is not new, but its implementation, especially in medicine, is a relatively novel [43]. The AVP may be the first untethered device capable of providing DR in a medically relevant and accurate context without the need for external hardware.

Notably, DR needs, in general, a VST capability and cannot be realized by OST displays, such as that in the HoloLens. The medical HMD from VOSTARS has addressed this by combining an OST display, that is, the view through a semitransparent glass, with a VST function [44]. VOSTARS has realized this with a semitransparent glass that can transition into a nontransparent mode. The AVP, by contrast, does not conform to the OST versus VST dichotomy but rather operates on the VST versus VR HMD spectrum, aligning with the reality-virtuality continuum, postulated by Milgram and Kishino in 1994 [45].

#### Virtual Eyes

Spooky at first sight, the outward display reveals a user’s eyes while wearing the AVP. According to Apple, this feature is meant to let others know when the AVP wearer is using apps or is completely immersed in a virtual world. This feature is not needed in OST AR devices, such as the HoloLens, because the headset wearer’s eyes can be seen through the transparent display. This feature is particularly crucial when considering patients who are dealing with serious illnesses. The transparency ensures that medical professionals, while using the device, do not create an additional layer of detachment by being obscured behind a screen, potentially alleviating concerns about the impact on patient-physician interactions.

#### 3D Camera

The AVP features a 3D camera, which allows a user to capture spatial photos and spatial videos in 3D. This could hold significant potential for the professional health care sector. The ability to capture spatial photos and videos in 3D could prove immensely valuable for documenting medical interventions. This is certainly also of interest for medical training, allowing residents to review and reenact treatments in 3D from different viewpoints. It could also serve as a tool to uncover malpractice. The 3D capabilities can also provide an alternative or an addition to the 3D documentation of crime and crash scenes, which is common practice during forensic and medicolegal investigations [46].

#### Interaction

According to Apple, a user can interact with virtual content by simply looking at an element, tapping the fingers together to select it, and using the virtual keyboard or dictation to type. There also seems to be a “visual search” function, which may be similar to the “visual look up” feature found on iPhones and iPads. “Visual search” will allow users to interact with items and text for opening webpages and translating into other languages in real time. Interestingly, this was already described by Sutherland [1] as “the language of glances” to interact with a computer, with an example where looking at a corner of a triangle makes it become rounded. Looking something up is definitely of interest, for example, to surgeons who have their hands full with (surgical) instruments or nonverbal users.

#### Optic ID

The AVP is supposed to support the optical identification of a user for authentication, such the iPhone’s face identification. The biometric method is enabled by scanning the iris of the user wearing the headset. This is not unique to the AVP because the HoloLens 2 already supported an iris authentication. However, this is also an important feature for the AVP in health care, where you have sensitive patient data, and unlocking the device and accessing the data should be permitted only for authorized users. Furthermore, it enables hands-free log-in in a potentially sterile environment.

#### ZEISS Optical Inserts

Carl Zeiss AG is working together with Apple to provide precision optics for users who require vision correction. This will improve the visual experience and provide greater comfort, as users do not need to wear their prescription glasses and frames inside the limited space within the AVP. In addition, it may help avoid side effects such as “overheating,” headaches, and light bleeding. The additional lenses can be attached magnetically to the main lens, which makes insertion and replacement easy.

#### Head Strap

The head strap of the AVP seems to be easily exchangeable, and there are already several alternatives (in terms of materials, colors, etc) available on the web. This will ultimately enable more comfort in comparison to the HoloLens and the Varjo XR-3, which are adjustable but still consist of quite stiff (plastic) frames around the head. It will definitely be more comfortable than the Sword of Damocles, developed by Ivan Sutherland and his student Bob Sproull [47], where the user had to be strapped into the AR or VR HMD device, which was suspended from the ceiling because it was so heavy. The head strap also contains the AVP’s speaker, which is placed directly over the user’s ears and is supposed to virtualize surround sound. However, the strap means that all main components are integrated with the visor. The HoloLens 2, for example, distributes the weight more evenly by integrating components in a plastic case located at the rear, including the battery and system on a chip board. This was done by Microsoft based on its experiences with the HoloLens 1 to make the HoloLens 2 more comfortable. On the contrary, the AVP seems to be worn more like a VR device that fits tightly onto the face instead of partly hovering in front of the user’s eyes, such as other AR devices that are more similar to prescription glasses. The AVP has a similar weight range (600-650 g) to other popular AR and VR headsets, even though the battery is not integrated into the device and is separate. In part, this could be attributed to the material, for example, the aluminum alloy frame. Furthermore, in a medical context, it is usually necessary to be able to easily disinfect the equipment used. A special version with a disinfectable head strap could meet this requirement.

#### 3D Persona Avatars

During the setup of the AVP, the user’s face is scanned by the headset to create a photorealistic avatar, which is then used by the device’s operating system, called VisionOS. For better fitting purposes, the user’s face can also be scanned with the TrueDepth camera of an iPhone, as seen at a public demo at the Worldwide Developers Conference in June 2023. Furthermore, the user’s ears can be scanned to optimize the speakers that are inside of the head strap because, aside from the shape and size of the head, the ear sizes, positions, and distances can vary a lot between users. All these user-specific options will make the headset more comfortable to wear, and this will finally increase the acceptability of the new device, not only in the health care sector but also in other sectors.

#### Unity

The Unity engine already supports pretty much all common headset devices, whether it be AR, VR, or MR, such as the Oculus (Palmer Luckey), HoloLens, or Google VR (Google LLC). Unity also supports the OpenVR application programming interface that has already been used in medicine [48]. Therefore, the support of the Unity engine of the AVP makes the development and porting of apps much easier. This also makes a comparison between and an evaluation of devices with the “same” app easier. Notably, Unity currently already allows the development of apps for iPhone, iPad, and Mac while deployment occurs via the Apple app store or Xcode, suggesting future development for the AVP. Overall, Unity support will enable faster development of apps, hopefully fast enough to reach a critical mass before end users may turn their backs, which is needed for the long-term success of the new Apple device.

#### Medical Device

Talking about the health care sector, we need to mention that the AVP is not certified as a medical device yet. We are not aware of whether Apple has plans in this direction, at least not publicly. Apple, however, promoted Complete HeartX as an education app for medical students by providing hyperrealistic 3D models and animations of the heart and medical issues. In comparison, Microsoft advertised the HoloLens 2 specifically for medical scenarios, for example, with a plenary presentation by Bernard Kress (who was at that time Principal Optical Architect, HoloLens team, Microsoft Corp) at the International Society for Optics and Photonics Medical Imaging in February 2020. Nevertheless, we are sure that the AVP will be used by researchers and companies for medical applications, similar to other devices, if Apple does not explicitly prohibit this somehow, for example, by monitoring their devices and locking them. The integration of the AVP with other software and hardware commonly used in clinical settings, for example, integration with electronic health records, will be interesting [49,50].

### First Experience

**Figure 1:**
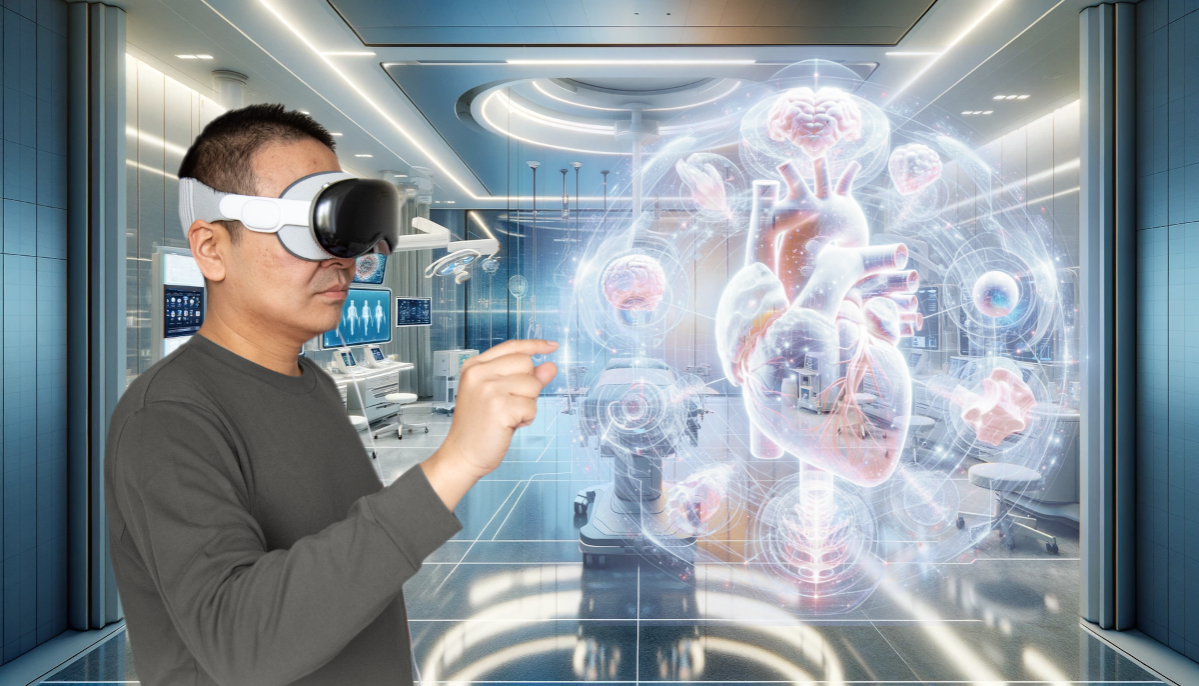
The illustration of a possible Apple Vision Pro scenario with one of the authors.

**Figure 2:**
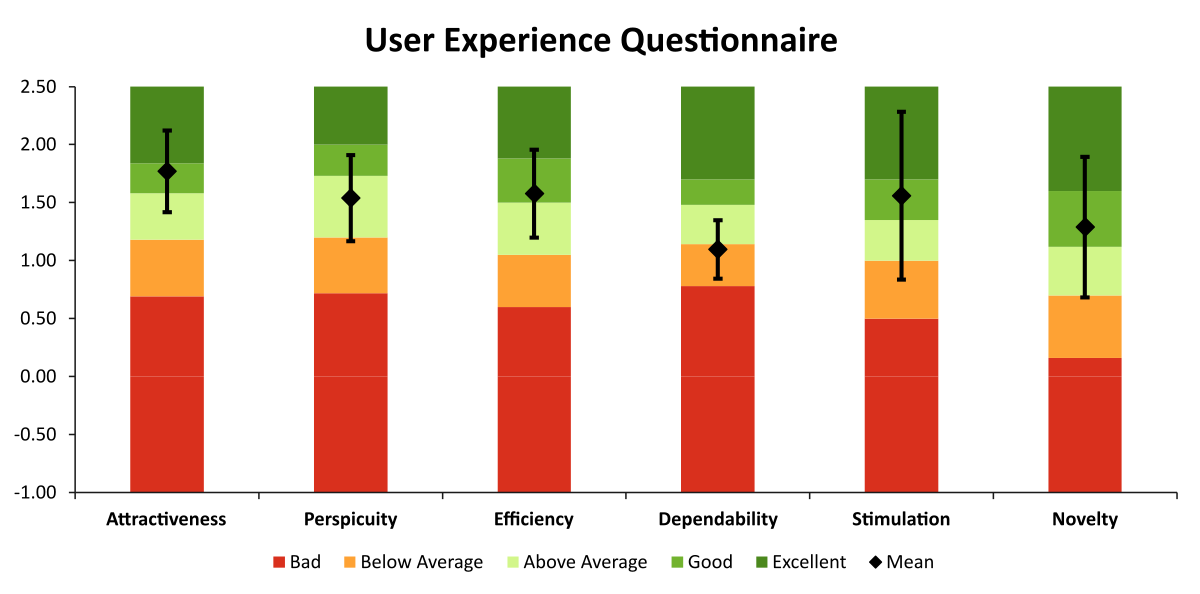
The results (means and SDs) of the User Experience Questionnaire (UEQ) regarding the attractiveness, perspicuity, efficiency, dependability, stimulation, and novelty of the Apple Vision Pro compared to those of a benchmark (bar plot in the background).

In March 2024, we had the opportunity to try out the AVP ourselves for the first time (Figure 1). We, therefore, conducted a standardized expert survey among colleagues (and ourselves) at our institute (the Institute for Artificial Intelligence in Medicine). The inclusion criterion was an experience of at least 10 minutes with the AVP. After obtaining written consent, we conducted a structured survey on the AVP. We collected baseline data from the respondents including experience with HMDs (Table 1), as well as asked Likert questions on how far the AVP corresponds to an “Ultimate Display” (Table 2). We also administered the User Experience Questionnaire (UEQ; Figure 2) as well as asked open-ended questions about what is missing from an “Ultimate Display” (Textbox 2).

**Table 1:** Characteristics of the experts interviewed (N=13).

Parameter		Value
**Sex**		
	Female	3 (23)
	Male	10 (77)
Age (y), mean (SD; range)		30.9 (6.5; 23-48)
Work experience (y), mean (SD; range)		4.3 (5.6; 0-20)
**Previous experience with HMDs^a^**		
	Yes	9 (69)
	No	4 (31)
**Previous experience with HMDs (hours)**		
	Mean (SD)	213.3 (400.8)
	Median (range)	1.0 (0-1000)

^a^HMD: head-mounted display.

**Table 2:** Likert questionnaire (N=13).

	Likert item (1=disagree; 7=agree)	Value, mean (SD)
1.	The Apple Vision Pro comes very close to an “Ultimate Display” in terms of realism.	5.5 (1.3)
2.	The Apple Vision Pro comes very close to an “Ultimate Display” in terms of interactivity.	5.0 (1.1)
3.	The Apple Vision Pro is more comfortable than other HMDs^a^.	4.9 (1.5)
4.	The Apple Vision Pro is more user-friendly than other HMDs.	5.1 (1.9)
5.	The Apple Vision Pro surpasses other HMDs in immersion and sense of presence.	5.4 (1.6)
6.	The Apple Vision Pro represents a significant technological advance.	5.7 (1.4)

^a^HMD: head-mounted display.

**Open questionnaire summary.**

**Negatives**

- Suboptimal weight distribution
- Comparatively heavy among head-mounted display (HMDs)
- Detachable light seal cushion poses potential damage risks and necessitates screen touching before and after use
- Requirement for specialized prescription glasses
- Short battery life (2 hours under normal use)
- Forced interaction method with eyes and pinch
- Inconsistent functionality of eye navigation near the borders of the field of view
- The absence of intelligent automatic transition between real and virtual worlds via the crown button
- Limited field of view compared to other HMDs
- Blurry resolution of real-world pass-through, hindering text legibility
- Clear differentiation between virtual and real environmental components by users

**Positives**

- Lightweight compared to the currently used hardware and materials
- Natural interaction via outward sensors
- Effective eye-tracking for seamless user interaction
- Rapid and simple adjustment of glasses for user interpupillary distance
- Innovative crown button design with wheel and push functionalities
- Smooth transition between real and virtual worlds facilitated by the crown button
- High-resolution internal display for realistic virtual objects
- High-quality spatial sound experience
- Seamless visualization of the real world
- Minimal app start-up and file loading times
- Autofocus and person blending and unblending for safety precautions
- Simple and fast to learn

Responses to the Likert questions on whether the AVP comes close to an “Ultimate Display” comprised values between 4.9 and 5.7 on a scale of 1 (disagree) to 7 (agree). The UEQ showed good mean results for attractiveness (1.77, SD 0.65), efficiency (1.58, SD 0.70), stimulation (1.56, SD 1.33), and novelty (1.29, SD 1.11) but above average mean result for perspicuity (1.54, SD 0.68) and below average mean result for dependability (1.10, SD 0.46) compared to a benchmark. This fits in with the general perception (Textbox 2) that the AVP is a significant improvement over other HMDs but still behind the “Ultimate Display.”

## Discussion

To summarize, we see Apple entering the MR headset market with its own device very positively. Another company’s contribution can benefit the technical progress in this area, not only from a hardware perspective but also from a software perspective, by enabling new user input concepts and apps. With the current hardware specifications and Apple’s history of delivering cutting-edge devices and beyond, there is a very good chance of another AR “evolution” or even “revolution” (Table 3). This is a very important aspect because (1) AR devices are sparse in comparison to VR devices, and (2) the product life cycle to a possible successor is in the range of years, which feels like ages when compared to the current pace of technological developments, especially in (medical) computer science [51]. For example, the first version of the HoloLens was introduced by Microsoft in 2016, and it took Microsoft >3 years to introduce its successor, the HoloLens 2 (released in limited numbers on November 7, 2019).

**Table 3:** Side-by-side comparison of the specifications of several existing mixed reality headsets with those of the upcoming Apple Vision Pro [52].

Parameter	Apple Vision Pro	Pico 4	Meta Quest 3	HTC Vive XR Elite	Varjo XR-3	HoloLens 2	Magic Leap 2
AR^a^	VST^b^	VST	VST	VST	VST	OST^c^	OST
VR^d^	Yes	Yes	Yes	Yes	Yes	No	No
Resolution (per eye)	3800×3000	2160×2160	2064×2208	1920×1920	1920×1920 focus and 2880 ×2720 peripheral	2048×1080	1440×1760
Refresh rate (Hz)	100	90	120	90	90	120	60
Visible field of view	Estimate 100 horizontal	104 horizontal and 103 vertical	110 horizontal and 96 vertical	102.13 horizontal and 91,27 vertical	115 horizontal and 90 vertical	43 horizontal and 29 vertical	44 horizontal and 53 vertical
Camera	Dual 6.5 MP^e^ pass-through cameras	16 MP RGB^f^ camera	Dual 18 PPD^g^ color pass-through cameras	16 MP RGB camera	Dual 12 MP 90 Hz cameras	Native pass-through 8 MP camera 30 Hz	Native pass-through 12.6 MP 60 fps RGB camera
Tracking type	6 DoF^h^ inside-out tracking via 12 integrated cameras; includes depth sensor and LIDAR^i^	6 DoF inside-out tracking	6 DoF inside-out tracking via 4 integrated cameras; includes depth sensor	6 DoF inside-out tracking via 4 integrated cameras; includes depth sensor	6 DoF marker-based tracking and non–marker-based tracking; includes LiDAR sensor	6 DoF inside-out tracking via 4 integrated cameras	6 DoF inside-out tracking
Tracking	Eyes, face, and hands	Hands	Hands, upper body, and leg position	Hands	Eyes and hands	Eyes and hands	Eyes and hands
Depth sensing	LiDAR+TrueDepth	Depth camera	Depth camera	Depth camera	LiDAR+RGB fusion	ToF^j^ depth	iToF^k^ depth
CPU^l^	Apple M2 and Apple R1	Qualcomm Snapdragon XR2	Qualcomm Snapdragon XR2 Gen 2	Qualcomm Snapdragon XR2 Gen 2	None	Qualcomm Snapdragon 850	Quad-core Zen2
GPU^m^	Apple M2 10-core GPU	Adreno 650	Adreno 740	Adreno 650	None	Adreno 630	AMD GFX10.2
RAM	Up to 16 GB	8 GB	8GB	12 GB	None	4 GB	16 GB
Storage	64 GB	128 or 256 GB	128, 256, and 512 GB	128 GB	None	64 GB	256 GB
OS^n^	visionOS	PICO OS 5.0 (Android)	Android	Android	None	Universal Windows Platform	Lumin OS
SDK^o^	visionOS	PICO Unity Integration SDK and OpenXR	Meta XR All-in-One SDK and OpenXR	Wave andOpenXR	Varjo Native and OpenXR	Windows Mixed Reality platform and OpenXR	Magic Leap and OpenXR
Battery life	2 hours	3 hours	2 hours	2 hours	Wired	3 hours	3.5 hours
Weight	600 g + headband 50 g + external battery 353 g	295 g + headband 291 g	509 g	625 g	594 g + headband 386 g	566 g	260 g
Untethered	Yes	Yes	Yes	No	No	Yes	Yes
Price (US $)	3499	430	499	1100	6500	3500	3299

^a^AR: augmented reality.

^b^VST: video see-through.

^c^OST: optical see-through.

^d^VR: virtual reality.

^e^MP: megapixel.

^f^RGB: red, green, blue.

^g^PPD: peak pixel density.

^h^DoF: degrees of freedom.

^i^LIDAR: light detection and ranging.

^j^ToF: time-of-flight.

^k^iToF: indirect time-of-flight.

^l^CPU: central processing unit.

^m^GPU: graphics processing unit.

^n^OS: operating system.

^o^SDK: software development kit.

Steve Jobs released the iPhone in 2007, creating a new category of smartphones. What made it special was that Apple combined existing technologies and software in a manner that made previously unexpected things possible, which Apple repeated with the iPad in 2010 and then with the Apple Watch in 2015. Hence, the AVP could lead to the breakthrough of not only AR, VR, or MR but also DR (because it is a pass-through technique) due to its enormous technological progress. Moreover, technological progress can not only enable more precise, patient-specific treatments but also make these treatments more efficient, for example, through hands-free, instant authentications; documentation; and the visualization of search queries and their results directly within the field of vision of the HMD user. Further, well-engineered AR can help overcome communication barriers in a face-to-face manner. Thus, it supports clinicians in a range of essential tasks and reduces the administrative burden on clinicians to allow them to spend more time with their patients, which is a key challenge for upcoming artificial intelligence (AI)-based foundation models [53].

Moreover, the recent rise of large language models [54], such as ChatGPT, cannot be ignored in the context of the upcoming AVP because it changed how we communicate with computers, and the AVP can certainly be enhanced with the integration of a ChatGPT-like bot. Even though ChatGPT is still in its infancy, when it comes to hard facts, especially in health care [55,56], it already enables a fluent, partly fact-based, conversation. This will, in the short or long run, also completely enter the headset market, where someone can discuss upcoming treatment steps on a humanlike level based on a trained large language model. However, we do not think that this will be specific to the AVP, and with ChatGPT, Microsoft seems to currently have the edge.

Another application is the metaverse, which integrates physical reality and VR [8]. The idea is that users and their avatars interact in an environment with access to an unlimited amount of health data. Metaverse applications focus primarily on AI-based medical practice and medical image–guided diagnosis and therapy [8]. The AVP could make an important contribution as a hardware enabler that allows users to move and interact in the metaverse in a more intuitive manner.

Nevertheless, digitization holds promise for making medical care more efficient. But will screens come between doctors and patients [57]? At the same time, for every hour doctors spend with their patients, they spend 2 hours in front of the computer [58]. The AVP’s untethered, external battery, and spooky-eye design with improved sensor and computing hardware has the potential to help health care professionals improve efficiency and patient outcomes, allowing more time for patient care. The improved hardware should allow the levels of accuracy and computing needed for the device to truly function as a stand-alone device for surgery or preoperative planning. Integration with patient records combined with AI allows for patient recognition and provides the health care professional with accurate data retrieval and documentation. The provision of guidance for devices and medical procedures can occur naturally through its outward-facing sensors, making reading books a thing of the past. The VST design opens up futuristic possibilities for DR, while the ghostly eyes maintain a sense of patient contact. We recognize that this is an optimistic outlook and will require the combined efforts of experts in the field.

Whether the AVP will blur the line between patient encounters and computer time also depends on patient and physician acceptance. In ophthalmology, the AVP is seen as a breathtaking application [59]. However, this must first be confirmed in clinical trials with rigorous scientific methodology and hard clinical end points. When the AVP is used for patient scenarios, the focus must be on the patient, not the technology, which is always a vehicle for existing problems.

## Conclusions

To sum up, the AVP enriches the current headset market and provides another alternative, especially in the limited AR segment. The AVP will likely be at the technological forefront in this sector, which puts pressure on other vendors to compete by drawing level with and surpassing Apple. The price is certainly a burden for entertainment consumers, but for professionals, especially in the health care sector, with the Microsoft HoloLens being in the same price range and the Varjo XR-3 costing twice as much, it is not a deal-breaker. This becomes even more *irrelevant* if seen in relation to the other hardware costs in health care, such as those of navigation systems and imaging equipment (computed tomography and magnetic resonance imaging scanners), which can reach millions. In Sutherland’s [1] sense and our experience, it is not yet the Wonderland that Alice or the doctors (added by us) walked into, but perhaps a bit closer.

## Abbreviations

AI: artificial intelligence
AR: augmented reality
AVP: Apple Vision Pro
DR: diminished reality
HMD: head-mounted display
MR: mixed reality
OST: optical see-through
VR: virtual reality
VST: video see-through
XR: extended reality
